# Liver infarctions as the first manifestation of antiphospholipid antibody syndrome in pregnancy: a case report

**DOI:** 10.1186/s13256-022-03324-8

**Published:** 2022-03-14

**Authors:** Claudia Meloni, Cornelia Schreiber, Jan-Dirk Studt, Simone Kamm, Manuela Di Chiara, Thomas Herren

**Affiliations:** 1 Department of Medicine, Limmattal Hospital, 100 Urdorferstrasse, 8952 Schlieren, ZH Switzerland; 2 Department of Medicine, University Hospital Basel, 4 Petersgraben, 4031 Basel, BS Switzerland; 3 Division of Medical Oncology and Hematology, University and University Hospital Zurich, 100 Raemistrasse, 8091 Zurich, ZH Switzerland; 4 Division of Gynecology and Obstetrics, Limmattal Hospital, 100 Urdorferstrasse, 8952 Schlieren, ZH Switzerland; 5 Department of Rheumatology, Winterthur Cantonal Hospital, 15 Brauerstrasse, 8400 Winterthur, ZH Switzerland; 6 University of Zurich, 71 Raemistrasse, 8006, Zurich, ZH Switzerland

**Keywords:** Pregnancy, Hepatopathy, Liver lesions, Abortion, Antiphospholipid antibody syndrome, Systemic lupus erythematosus, Case report

## Abstract

**Background:**

The differential diagnosis of abdominal pain in pregnant women is broad. Liver diseases as the origin of abdominal pain in pregnancy are rare, and severe forms occur in less than 0.1% of pregnancies. Some disorders, such as hemolysis, elevated liver enzymes, low platelets (HELLP) syndrome and preeclampsia, are unique to pregnancy, while others, such as antiphospholipid antibody syndrome, may manifest in pregnancy but have consequences beyond the current pregnancy. All of them require prompt identification and treatment.

**Case presentation:**

A 27-year-old Caucasian woman who was 15^+1^ weeks pregnant reported to the emergency department twice due to stabbing right-upper-quadrant abdominal pain. Initial laboratory testing revealed mild leukocytosis and slightly elevated liver enzymes. On second presentation, the patient was febrile and had an increased C-reactive protein concentration. Over the course of the next days, nonhemolytic anemia and thrombocytopenia emerged with elevated liver enzymes. Coagulation studies also revealed a prolongation of activated partial thromboplastin time. Magnetic resonance imaging showed nonspecific alterations in the right liver lobe, possibly corresponding to infection or infarction. A hepatic viral infection was ruled out. At that time, the most likely diagnosis was cholangitis with liver abscess formation, and antibiotic therapy was started. Further worsening of the anemia and thrombocytopenia, development of proteinuria, together with a miscarriage on the fourth day of hospitalization resulted in the tentative diagnosis of (triple-positive) antiphospholipid antibody syndrome, which was confirmed 12 weeks after the initial investigation. Treatment consisted of prompt anticoagulation with heparin and later on with a vitamin K antagonist as well as high-dose glucocorticoid therapy. There was no need for intravenous immunoglobulin therapy or plasma exchange, although we suspected a catastrophic form of antiphospholipid antibody syndrome due to infarctions of the liver, placenta, and possibly kidneys (proteinuria). The outcome was favorable.

**Conclusion:**

We report a 27-year-old pregnant woman whose abdominal pain was caused by liver infarctions as the first manifestation of catastrophic antiphospholipid antibody syndrome. The antiphospholipid antibody syndrome was possibly secondary to hitherto clinically silent systemic lupus erythematosus since the antinuclear antibodies were increased later on. Hydroxychloroquine therapy was initiated to prevent antiphospholipid antibody syndrome recurrence in a future pregnancy.

## Background

Physicians examine patients with abdominal pain quite often, and the differential diagnosis is broad. In cases involving pregnant women, diseases unique to pregnancy must be considered. Liver diseases are rare, and severe forms occur in less than 0.1% of pregnancies [1]. Thrombotic microangiopathies such as hemolysis, elevated liver enzymes, low platelets (HELLP) syndrome, preeclampsia, thrombotic thrombocytopenic purpura, atypical hemolytic uremic syndrome, and antiphospholipid antibody syndrome (APS) are among them. Their prompt recognition and treatment are essential. Infarctions of the liver seldom occur because of its dual blood supply [2]. However, liver infarctions have been reported in the context of APS [3, 4]. Here, we describe the case of a pregnant woman with abdominal pain due to liver infarctions. The unusual presentation, including the diagnostic dilemma and the complex therapeutic options, is discussed, and the ultimate diagnosis of catastrophic antiphospholipid antibody syndrome (CAPS) and its therapy are reviewed.

## Case presentation

A 27-year-old, previously healthy Caucasian woman who was 15^+1^ weeks into her first pregnancy presented to the emergency room with stabbing right-upper-quadrant abdominal pain of a few days’ duration. The pain did not increase depending on respiration or posture. An abdominal ultrasound confirmed an intact singleton pregnancy, and the findings were otherwise unremarkable. Laboratory tests revealed mild leukocytosis and slightly elevated liver enzymes but no proteinuria (Table 1). The patient was discharged home with a prescription for acetaminophen. However, the pain increased and was accompanied by nausea and vomiting, prompting another visit to the emergency room the following week. The patient was afebrile (36.5 °C) and normotensive (135/86 mmHg). Her heart rate was 69/minute, and there were no signs of heart failure. Her right upper abdomen was tender with no guarding. The remaining physical including the neurological examination was normal. Again, laboratory analyses showed mild leukocytosis and an increase in liver enzymes and C-reactive protein (Table 1). The patient was admitted for further diagnostic workup. Her past medical history, including the gynecological and obstetric history, was quite unremarkable, except for an appendectomy. In particular, she and her next of kin had no arterial or venous thromboembolism or rheumatic disease prior. She developed urticaria in response to mefenamic acid, a nonsteroidal antiinflammatory drug. Before becoming pregnant, she did not take any medication, but she smoked 10 cigarettes per day. A multivitamin drug including folic acid and iron was prescribed at the beginning of pregnancy. She did not consume alcohol or illicit drugs. The patient was married and worked as a retail assistant.

**Table 1:** Laboratory studies

Parameter	Patient value, day 1 (ER)	Patient value, day 6 (admission)	Patient value, day 9 (abortion)	Patient value, day 17 (discharge)	Reference range	Units
**Plasma**						
Leukocytes	11.6	11.3	17.2	12.3	4.0–9.8	G/L
Hemoglobin	137	124	90	101	123–153	g/L
Thrombocytes	146	175	38	82	> 150	G/L
INR	–	1.3	1.1	1.2	≤ 1.1	
aPTT	–	124	145	118	25–36.5	Seconds
Fibrinogen	–	9.1	9.9	4.9	1.8–3.5	g/L
Haptoglobin	–	–	2.63	–	0.3–2.0	
CRP	5	31	150	24	< 5	mg/L
Creatinine	140	43	41	63	44–80	µmol/L
ASAT	75	66	114	61	< 35	U/L
ALAT	72	175	167	107	< 35	U/L
Bilirubin	10	9	10	–	< 21	µmol/L
Alkaline phosphatase	55	89	97	179	35–104	U/L
Gamma glutamyl transferase	26	54	75	229	< 40	U/L
**Urine**						
Protein (dipstick)	Negative	Negative	–	0.25	Negative	g/L
Total protein	–	–	651	–	< 140	mg/24 hour

*ER* = emergency room, *INR* = international normalized ratio, *aPTT* = actived partial thromboplastin time, *CRP* = C-reactive protein, *ASAT* = aspartate aminotransferase, *ALAT* = alanine aminotransferase

Inflammation markers and liver enzymes continued to increase over the next few days. Normocytic, normochromic anemia without signs of hemolysis (positive direct antiglobulin test [anti-IgG and anti-C3d] but normal haptoglobin and bilirubin concentrations) and mild-to-moderate thrombopenia emerged. Coagulation studies showed marked prolongation of the activated partial thromboplastin time (aPTT) to over 100 seconds; however, the prothrombin time was normal, and the fibrinogen concentration increased (Table 1). There were no bleeding signs clinically. Esophagogastroduodenoscopy combined with endosonography ruled out cholecystitis, cholecysto- and choledocholithiasis, and a dilated biliary tract. Abdominal MRI showed nonspecific alterations in the right liver lobe, possibly corresponding to infection or infarction, splenomegaly, and peripancreatic lymphadenopathy. No valvular lesions were detected by transthoracic echocardiography. No infection with hepatitis A, B, C, D, or E viruses, human immunodeficiency virus (HIV), severe acute respiratory syndrome coronavirus 2 (SARS-CoV-2), herpes simplex virus (HSV), parvovirus B19, cytomegalovirus (CMV), or Epstein–Barr virus (EBV) was found.

At this time, cholangitis complicated by liver abscess formation was considered the most likely diagnosis of the febrile patient (39.1 °C), and antibiotic treatment with amoxicillin/clavulanate 2.2 g intravenously tid was initiated, and after 3 days escalated to piperacillin/tazobactam 4.5 g intravenously tid for a total of 10 days. The vitality of the fetus had been confirmed daily. However, hydrops fetalis and intrauterine growth retardation were detected by ultrasound on the third day after admission.

Increasing abdominal pain required treatment with opioids: oxycodone 10 mg plus naloxone 5 mg orally tid and morphine 2 mg intravenously prn. Anemia and thrombocytopenia worsened, and inflammatory markers and liver enzymes increased (Table 1). Miscarriage occurred on the fourth day of hospitalization, corresponding to 15^+4^ gestational weeks, and labor was induced with sulprostone 240 µg/hour intravenous. After expulsion, the patient received a loading dose of 5 g magnesium sulfate intravenous, followed by a continuous intravenous infusion of 16 g magnesium sulfate per day for seizure prophylaxis, since preeclampsia was considered possible at this time. The growth-retarded fetus had no morphological abnormalities otherwise. Transabdominal chorionic villus sampling, which had been performed the preceding day, did not reveal fetal chromosomal aberrations.

**Fig. 1:**
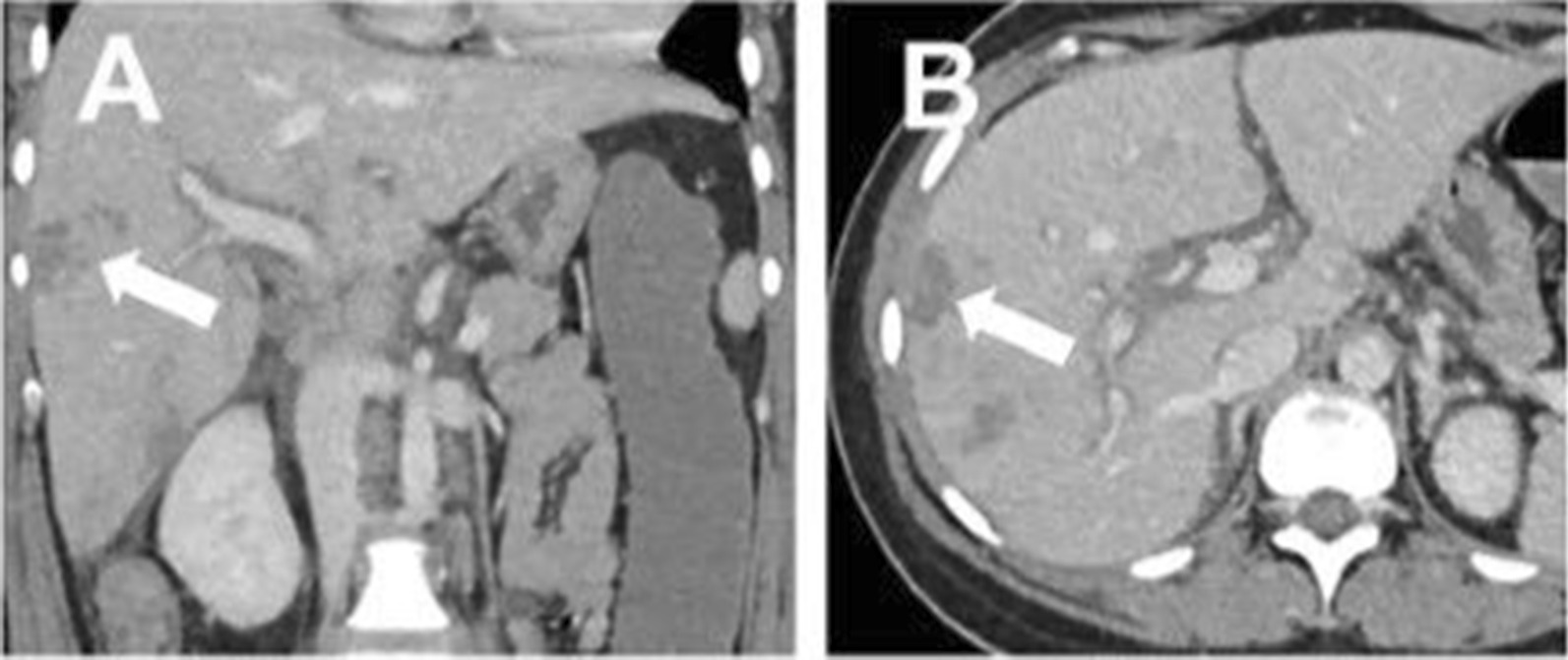
Representative images of an abdominal CT scan. Frontal (**A**) and transverse planes (**B**). There were multiple liver lesions, with the largest measuring 3 cm in diameter (arrows). *CT scan* = computed tomography scan

Histopathological examination of the placenta, in part removed by curettage, showed a maternal intervillous circulatory disorder with infarctions extending to 15% of its volume. Petechial bleeding was seen on the liver by diagnostic laparoscopy, and a CT scan documented worsening of the liver lesions, including periportal edema (Fig. 1). Moreover, small bilateral pleural effusions were present.

Additional blood coagulation tests showed the presence of lupus anticoagulant (dilute Russel viper venom time [dRVVT] ratio of 3.3 [normal range < 1.3]), and anti-cardiolipin IgG and IgM antibodies increased to 55 U/ml and 61 U/ml (normal < 40 U/ml), respectively. Anti-β2 glycoprotein IgM antibodies were elevated as well (35 U/ml, normal < 10 U/ml). Antinuclear antibodies (ANAs) or autoantibodies relevant for autoimmune hepatitis were not present.

## Discussion and conclusion

We report a woman with abdominal pain during pregnancy. Based on abdominal MRI findings and initial laboratory workup, a diagnosis of cholangitis with liver abscess formation was made, and antibiotic treatment was started. This diagnosis had to be revoked after intrauterine death of the fetus, histologically confirmed substantial infarction of the placenta, proteinuria, consistently prolonged aPTT, presence of lupus anticoagulant, and positive APS antibodies. The first manifestation of CAPS with a gestational complication including liver infarctions and renal involvement is unique and difficult to diagnose. Moreover, the jury is still out regarding whether the patient has a primary CAPS or whether it is secondary to hitherto clinically silent systemic lupus erythematosus (SLE).

The differential diagnosis of pregnancy-associated liver disease includes thrombotic microangiopathies such as HELLP syndrome or thrombotic thrombocytopenic purpura, preeclampsia, intrahepatic cholestasis, acute fatty liver of pregnancy (AFLP), and hyperemesis gravidarum. Since hemolysis was not present, thrombotic microangiopathy was deemed unlikely, as was AFLP, which typically occurs at a later stage of pregnancy. There was no hyperemesis. The patient reported no itching, which made intrahepatic cholestasis an unlikely diagnosis. Preeclampsia was ruled out because of normal blood pressure, even though thrombocytopenia, proteinuria, and elevated liver enzymes are frequently observed in this condition. Angiogenic factors, whose quantification may assist in ruling out preeclampsia, were not measured [5, 6]. The initial diagnosis of cholangitis with hepatic abscess was based only on MRI findings and routine laboratory data.

Miscarriage in the 16th week of gestation, the consistently prolonged aPTT, and the detection of lupus anticoagulant as well as elevated anticardiolipin and anti-β2 glycoprotein antibodies led us to assume (triple-positive) APS. This diagnosis was confirmed by persistence of the lupus anticoagulant and high titers of anti-cardiolipin and anti-β2 glycoprotein antibodies 12 weeks after the initial investigation.

APS is a systemic autoimmune disease characterized by thrombotic and/or obstetric manifestations in patients with persistent antiphospholipid antibodies. The diagnostic criteria of APS according to the revised Sapporo classification are shown in Table 2. Thrombosis may be venous, arterial, or microvascular [7]. In our patient, the findings of the MRI and CT scans were consistent with liver infarctions, although abdominal manifestations of APS are uncommon. Of these, liver involvement is the most frequent. Liver pathologies in APS may include thrombotic diseases, such as Budd–Chiari syndrome, hepatic veno-occlusive disease (HVOD), and liver infarctions, and nonthrombotic diseases, such as cirrhosis, autoimmune hepatitis, and portal hypertension [2].

**Table 2:** Revised Sapporo classification criteria for antiphospholipid antibody syndrome (APS) [8] Adapted from Miyakis *et al*. 2006

APS is present if at least one clinical and one laboratory criterion is met:	
Clinical criteria	Laboratory criteria
1. Vascular thrombosis	1. Lupus anticoagulant (LA)^a^
≥ 1 clinical episode of arterial, venous or small vessel thrombosis in any tissue or organ	Present in plasma, on ≥ 2 occasions at least 12 weeks apart
2. Pregnancy morbidity	2. Anti-cardiolipin (aCL) antibody
≥ 1 unexplained death of a morphologically normal fetus at or beyond the 10th week of gestation with normal fetal morphology documented by ultrasound or direct examination	IgG and/or IgM isotype in serum or plasma, present at medium or high titer, on ≥ 2 occasions at least 12 weeks apart
≥ 1 premature birth of a morphologically normal neonate before the 34th week of gestation due to eclampsia or severe preeclampsia or placental insufficiency	3. Anti-β2-glycoprotein-I antibody
	IgG and/or IgM isotype in serum or plasma on ≥ 2 occasions at least 12 weeks apart
≥ 3 unexplained consecutive spontaneous abortions before the 10th week of gestation	

^a^False-positive LA results may occur in patients treated with warfarin, heparin, or direct oral anticoagulants

*APS* = antiphospholipid antibody syndrome, *LA* = lupus anticoagulant, *aCL* = anti-cardiolipin antibody

Obstetric APS manifestations include fetal loss after the 10th week of gestation, recurrent early miscarriage, intrauterine growth retardation, and severe preeclampsia [7].

In addition, atypical clinical manifestations of APS, including cutaneous (for example livedo racemosa), pulmonary (for example, pulmonary arterial hypertension or alveolar hemorrhage), cardiac (for example, nonbacterial Libman–Sacks endocarditis), and neurological (for example, migraine or chorea) manifestations, may occur. A small subgroup of patients presents with livedoid vasculopathy and cerebral lesions known as Sneddon syndrome. Typical laboratory findings in APS are (hemolytic) anemia, thrombocytopenia, prolonged aPTT, and renal insufficiency, including proteinuria.

Macro- or microvascular thrombosis simultaneously affecting multiple organs or tissues (≥ 3 within 7 days) is a hallmark of CAPS [9], which is observed in approximately 1% of patients. CAPS is a life-threatening condition and is characterized by multiorgan dysfunction due to multiple thromboembolic incidents [10]. In our patient, we suspected CAPS with involvement of the liver, placenta, and possibly the kidneys (proteinuria).

APS is frequently associated with autoimmune diseases such as SLE, which may be the underlying cause of APS in approximately 35% of patients [11]. Although initially negative, the patient’s ANA titer increased to 1:1280 (normal < 1:320) one month after her initial presentation and persisted 3 months later, suggesting secondary APS as the first manifestation of possible SLE [12]. However, the patient had no other clinical symptoms or signs of SLE, such as fever, photosensitivity, aphthous ulcers, alopecia, or arthritis. In addition, her ANA immunofluorescence pattern was not typical for SLE, and antibodies to ds-DNA were not present. Anti-histone and anti-chromatine antibodies were slightly elevated (2.6 U [normal < 1.0 U] and 38 U/ml [normal < 20 U/ml], respectively) 6 months after the hospitalization [13]. Although suspicion of an underlying autoimmune disease is high, a definite diagnosis of SLE could not be established because symptoms or signs of SLE are absent even 1 year after the hospitalization. Moreover, clinical and immunological criteria which are consistent with the diagnosis of SLE (thrombocytopenia, pleural effusion, proteinuria, and APS antibodies) are more likely explained by the CAPS and the volume treatment in the ICU [12].

We initiated therapeutic-level anticoagulation with unfractionated heparin (heparin 25,000 U intravenous starting on day 5) and high-dose glucocorticoids (50 mg oral prednisone daily after an initial dose of 100 mg starting on day 4). Omeprazole 40 mg intravenous was administered as stress ulcer prophylaxis during the hospitalization. Normalization of liver enzymes and thrombocytopenia was achieved in due course. Due to this favorable response, the patient did not require plasma exchange or intravenous immunoglobulins, despite the suspected CAPS.

The patient was discharged without residual abdominal pain after 12 days. Her medication included tapering doses of oral prednisone (dose reduction by 5 mg every 2 weeks) and therapeutic-level low-molecular-weight heparin (dalteparin 15,000 U s.c. starting on day 11), which was transitioned to a vitamin K antagonist (phenprocoumon, each tablet containing 3 mg) with a target INR level of 2.5–3.0 for an indefinite duration starting 1 week after the hospitalization. Lifelong anticoagulation with a vitamin K antagonist is indicated; direct oral anticoagulants should be avoided, especially in triple-positive APS. Gynecological, hematological and rheumatological outpatient follow-up was scheduled. Future second pregnancy will require early substitution of the vitamin K antagonist with a therapeutic dose of low-molecular-weight heparin plus low-dose aspirin. Close gynecological, hematological, and rheumatological care remains of key importance during follow-up. To prevent a flare-up in a future pregnancy, and because of the remote possibility of SLE, hydroxychloroquine therapy was started at an initial dose of 200 mg bid orally 6 months after the hospitalization. The dose was tapered to 200 mg qd orally after 8 weeks, but will be increased to 200 mg bid again in case of a planned pregnancy. After 1 year, the health condition of the patient is excellent, and the only medications the patient takes regularly are phenprocoumon and hydroxychloroquine. The lupus anticoagulant is still present, as are the anti-cardiolipin IgG antibodies (48 U/ml). The anti-β2 glycoprotein antibodies disappeared in the meantime. She plans a future pregnancy.

In conclusion, the abdominal pain of the pregnant woman discussed was due to liver infarctions, most likely caused by thrombotic occlusion of liver artery branches. Two additional thrombotic manifestations were detected, namely infarction of the placenta (diagnosed after abortion) and possibly renal (proteinuria). Prolonged aPTT, positive lupus anticoagulant, and elevated anticardiolipin and anti-β2 glycoprotein antibodies were consistent with the diagnosis of CAPS. Lifelong anticoagulation with a vitamin K antagonist is indicated. If CAPS is diagnosed, high-dose glucocorticoid therapy, intravenous immunoglobulin therapy, and plasma exchange must be considered in addition to therapeutic anticoagulation. If APS is secondary to SLE, either hydroxychloroquine or low-dose corticoid therapy can be the therapeutic choice. Any further pregnancy requires close interdisciplinary collaboration.

## Data Availability

Not applicable.

## Abbreviations

HELLP: Hemolysis, elevated liver enzymes, low platelets
APS: Antiphospholipid antibody syndrome
CAPS: Catastrophic antiphospholipid antibody syndrome
aPTT: Activated partial thromboplastin time
ANA: Antinuclear antibodies
SLE: Systemic lupus erythematosus
AFLP: Acute fatty liver of pregnancy
HVOD: Veno-occlusive disease
LA: Lupus anticoagulant
aCL: Anti-cardiolipin antibody
